# Implementation of an antimicrobial stewardship program in the Vascular Surgery ward of a university tertiary care hospital in Pavia, Northern Italy

**DOI:** 10.1186/s12879-023-08061-x

**Published:** 2023-03-07

**Authors:** Marco Vecchia, Marta Colaneri, Paolo Sacchi, Lea Nadia Marvulli, Andrea Salvaderi, Jessica Lanza, Stefano Boschini, Franco Ragni, Piero Marone, Sara Cutti, Alba Muzzi, Carlo Marena, Monica Calvi, Luigia Scudeller, Enrico Maria Marone, Raffaele Bruno

**Affiliations:** 1 Division of Infectious Diseases Unit, Fondazione IRCCS Policlinico San Matteo, Pavia, Italy; 2 Division of Vascular Surgery Unit, Fondazione IRCCS Policlinico San Matteo, Pavia, Italy; 3 Microbiology and Virology Unit, Fondazione IRCCS Policlinico San Matteo, Pavia, Italy; 4 Medical Direction, Fondazione IRCCS Policlinico San Matteo, Pavia, Italy; 5 Pharmacy Unit, Fondazione IRCCS Policlinico San Matteo, Pavia, Italy; 6 Head, Research and Innovation Unit, IRCCS Azienda Ospedaliero-Universitaria di Bologna, Bologna, Italy; 7 Department of Medical, Surgical, Diagnostic and Paediatric Science, University of Pavia, Pavia, Italy

**Keywords:** Vascular surgery, Antimicrobial stewardship program, Multidisciplinary team

## Abstract

**Purpose:**

The commitment of multidisciplinary teams in antimicrobial stewardship programs (ASPs) is often inadequately considered, especially in surgical wards. We wanted to evaluate clinical, microbiological, and pharmacological outcomes before and after the implementation of an ASP in the Vascular Surgery ward of Fondazione IRCCS Policlinico San Matteo, a tertiary care hospital in Pavia, Italy.

**Methods:**

This was a quasi-experimental quality-improvement study. The antimicrobial stewardship activity was conducted twice a week for 12 months and consisted of both prospective audit and feedback of all the ongoing antimicrobial prescriptions by the infectious diseases’ consultants and educational meetings for the healthcare workers of the Vascular Surgery ward. For comparison between the study periods, Student t test (Mann–Whitney test for skewed distributions) was used for quantitative variables (ANOVA or Kruskall-Wallis for > 2 groups respectively), and Pearson’s chi-squared test (Fisher exact test where appropriate) for categorical variables. 2-tailed tests were used. P-value significance cut-off was 0.05.

**Results:**

During the 12-month intervention period, among a total number of 698 patients, 186 prescriptions were revised, mostly leading to de-escalating an ongoing antimicrobial therapy (39, 20.97%). A statistically significant reduction in isolates of carbapenem-resistant *Pseudomonas aeruginosa* (p-value 0.003) and the absence of *Clostridioides difficile* infections were reported. No statistically significant changes were observed in terms of length of stay and all-cause in-hospital mortality. A significant decrease in the administration of carbapenems (p-value 0.01), daptomycin (p-value < 0.01) and linezolid (p-value 0.43) was registered. A significant reduction in antimicrobial costs was also observed.

**Conclusions:**

The implementation of a 12-month ASP brought significant clinical and economic results, highlighting the benefits of a multidisciplinary teamwork.

## Background

It has currently become a commonly accepted wisdom that antimicrobial resistance (AMR) is a major threat to global health [1], and infections caused by multidrug resistant organisms (MDRO) are undoubtedly associated with increased morbidity, mortality and healthcare costs [2, 3]. Hence, since the causal role of antimicrobial use in the development of antimicrobial resistance is not questionable [4], antimicrobial stewardship programs (ASPs), aimed at optimizing antimicrobial consumption, are increasingly in demand.

However, a key point which appears to be as essential as rarely stressed, is the extent of multidisciplinary teams in achieving these interventions [5]. This means that it is not the infectious diseases (ID) specialist who merely imparts an over specific expertise; rather, the multidisciplinary stewardship team would include other specialists, who may take the occasion to become more aware of the AMR threat and consequently keen to improve the management of antimicrobial drugs in their wards of competence.

This topic applies not only to clinical wards, but also and especially to surgical ones, as for some of the most common surgical conditions, an infectious aetiology can be recognized [6]. Particularly in the Vascular Surgery ward, patients with chronic peripheral artery disease (PAD), often associated with insufficient glycaemic control and neuropathy are at increased risk of acquiring MDRO infections, thus frequently needing multiple antibiotic treatments [7]; moreover, surgical procedures expose them to surgical site infections.

With this in mind, we hereby sought to firstly evaluate how the implementation of an ASP based on a multidisciplinary team impacted on the occurrence of MDRO infections in the Vascular Surgery ward of our tertiary care hospital in Pavia, Northern Italy. Secondly, we evaluated how this ASP affected some hospital indicators of healthcare quality, such as the number of admissions in the Vascular Surgery ward for at least 48 h, the mean length of stay (LOS), the all-cause in-hospital mortality rate, the antimicrobial consumption, and the costs. Specifically, we selected readily recognisable measures, that are not only straightforward to evaluate before, during and after the intervention, but more importantly, potentially feasible for all surgical and non-surgical wards.

## Methods

### Study design, settings, and duration

This quasi-experimental quality-improvement study was applied in the Vascular Surgery ward of Fondazione IRCCS Policlinico San Matteo, a tertiary care hospital in Pavia, Italy.

The study was conceived and designed according to the SQUIRE 2.0 guidelines [8], with the aim to evaluate clinical, microbiological, and pharmacological outcomes by comparing a 12-month baseline period prior to the implementation of the ASP (Period A) to a 12-month period following its start (Period B). Specifically, Period A covered from the 1st July 2017 to the 30th June 2018, while Period B ranged from the 1st July 2018 to the 30th June 2019.

Data related to Period A for comparison were retrospectively collected from the hospital digital data warehouse, clinical records, and discharge letters. The monthly antibiotic and antifungal consumptions were inferred from the dispensation from the hospital pharmacy to the Vascular Surgery ward and converted in Defined Daily Dose (DDDs) per 100 patient days (PDs), according to the WHO definition.

### Intervention

Our antimicrobial stewardship intervention was based on the presence of at least two ID consultants in the Vascular Surgery ward twice a week (on Monday and Thursday afternoons) for about two hours per day and was continued for a 12-month period (Period B). It included two types of enabling elements:

1. *Prospective audit and feedback*. For every patient hospitalised for at least 48 h and receiving at least one antibiotic and/or antifungal drug for therapeutic purposes during the intervention activity, a revision of the antimicrobial prescription was conducted through an active discussion among the two ID consultants and a resident or a senior surgeon, resulting in a written consultation included in the medical record of the patient. Each evaluation considered the clinical picture, blood tests, radiological exams and microbiological results. Antimicrobials prescribed for surgical prophylaxis were not revised. Decisions on the prescriptions were coded as follows: *Antimicrobials not recommended; Stop antimicrobials; De-escalate antimicrobials* (by switching from parenteral to oral, narrowing the spectrum of activity or reducing the number of drugs administered); *Change antimicrobials; Change dosage of antimicrobials; Continue antimicrobials; Start antimicrobials; Escalate antimicrobials* (by switching from oral to parenteral, broadening the spectrum of activity, increasing the number of drugs administered).
2. *Educational meetings about antimicrobial stewardship and infection control***.** During Period B, monthly meetings were organized by the ID consultants to increase knowledge about AMR, hospital-acquired infections and infection control. Particularly, the consultants showed both medical and non-medical healthcare workers staff (i.e., nurses and auxiliary staff), the basic principles of patient contact isolation, cohorting, hand hygiene and the use of personal protection equipment (PPE) in case of patients either colonized or infected with MDRO or *Clostridioides difficile*.

### Measures

The primary outcome of the study was to evaluate the impact of the implementation of an ASP in the Vascular Surgery ward on the occurrence of MDRO infections.

Secondary outcomes included changes before, during and after the implementation of the ASP in terms of number of admissions in the Vascular Surgery ward for at least 48 h, length of stay (LOS), all-cause in-hospital mortality rate, antimicrobial consumption, and costs.

### Definitions

Definitions used in the paper and codes for the types of infections diagnosed in the Vascular Surgery ward during the study period are summarized in Table 1.

**Table 1:** Summary of definitions used

Definition		References and guidelines
Acute bacterial skin and skin structure infection (ABSSSI) and/or Surgical site infection (SSI)	ABSSSI: A bacterial infection of the skin with a lesion size area of at least 75 cm^2^ (lesion size measured by the area of redness, oedema and induration) SSI: An infection related to a surgical procedure that occurs near the surgical site within 30 days following surgery (or up to 90 days following surgery where an implant is involved)	FDA [9] CDC [10]
Bacteraemia	The presence of viable bacteria in the blood documented by a positive blood culture result	CDC [11]
Defined daily dose (DDD)	The assumed average maintenance dose per day for a drug used for its main indication in adults. For antimicrobial consumption in hospital, the indicator DDD/100 patients-days is generally used	WHO [12]
Diabetic foot infection (DFI)	Foot wound infection in diabetic patients defined by the presence of at least two classic findings of inflammation or purulent secretions	IDSA [13]
Hospital-acquired pneumonia (HAP)	Pneumonia acquired ≥ 48 h after hospital admission	IDSA [14]
Intra-abdominal infections (IAIs)	Infections that develop within the abdominal cavity, usually classified into uncomplicated and complicated	WSES [15]
Multidrug-resistant organisms (MDRO)	Microorganisms, predominantly bacteria, that are resistant to one or more classes of antimicrobial agents	CDC [16]
No infection/Colonisation	Absence of local or systemic signs and symptoms of infection, even in presence of bacterial isolation	
Sepsis	Life-threatening organ dysfunction caused by a dysregulated host response to infection	Singer et al., 2016 [17]
Septic arthritis and/or Spinal infections	Septic arthritis: An acute inflammation involving one or more joints on an infectious basis confirmed by a positive culture of synovial fluid Spinal infections: Include vertebral, discitis and spondylodiscitis	Colston at Atkins, 2018 [18] EANM/ESNR/ESCMID [19]
Urinary tract infections (UTIs)	Infections involving any part of the urinary system. They include both lower (cystitis) and upper (pyelonephritis) UTIs and are classified into uncomplicated and complicated	EAU [20]

### Statistical analysis

Descriptive statistics were produced for all variables. Mean and standard deviation (SD) are presented for normally distributed variables, and median and interquartile range (IQR) for non-normally distributed variables, numbers, and percentages for categorical variables. Whenever relevant, 95% confidence intervals (95% CI) were calculated. Shapiro Wilk’s and Kolmogorov–Smirnov test, as well as visual methods, were applied to test for normality.

For comparison between the study periods, Student t test (Mann–Whitney test for skewed distributions) was used for quantitative variables (ANOVA or Kruskall-Wallis for > 2 groups respectively), and Pearson’s chi-squared test (Fisher exact test where appropriate) for categorical variables. In all cases, 2-tailed tests were used. P-value significance cut-off was 0.05.

Stata computer software version 15.0 (Stata Corporation, 4905 Lakeway Drive, College Station, Texas 77845, USA) was used for statistical analysis.

## Results

Between the 1st July 2018 and the 30th June 2019, among a total number of 698 patients admitted in the Vascular Surgery ward for at least 48 h, 108 (15.67%) patients received at least one evaluation from the ID consultants, for a maximum of six different evaluations for a single patient. The median age was 71 (IQR 63–78) and 79 patients (73.15%) were males. The most prevalent underlying disease was of cardiovascular aetiology, followed by diabetes and metabolic comorbidities (Table 2).

**Table 2:** Characteristics of patients evaluated in the Vascular Surgery ward

Variables	Overall (N = 108)
Age (y), median (IQR)	71 (63–78)
Gender (male), n (%)	79 (73.15)
Comorbidities	
Cardiovascular, n (%)	102 (94.44)
Diabetes, n (%)	48 (44.44)
Haematologic, n (%)	5 (4.63)
HCV, n (%)	4 (3.70)
HIV, n (%)	1 (0.93)
Immunologic, n (%)	4 (3.70)
Metabolic, n (%)	59 (54.63)
Neurologic, n (%)	4 (3.70)
Oncologic, n (%)	3 (2.78)
Osteoarticular, n (%)	3 (2.78)
Respiratory, n (%)	15 (13.89)
Urologic, n (%)	17 (15.74)
Type of infection evaluated	
ABSSSI and/or surgical site infection, n (%)	52 (48.15)
Bacteremia, n (%)	2 (1.85)
Diabetic foot infection, n (%)	10 (9.26)
Hospital-acquired pneumonia, n (%)	15 (13.89)
Intra-abdominal infection, n (%)	7 (6.48)
No infection/colonization, n (%)	13 (12.04)
Sepsis, n (%)	1 (0.93)
Septic arthritis and/or osteomyelitis, n (%)	6 (5.55)
Urinary tract infection, n (%)	2 (1.85)

Overall, 186 prescriptions were revised. The most prevalent infectious condition evaluated during the intervention period was ABSSSI and/or surgical site infection, accounting for 52 patients (48.15%), followed by HAP (15 patients, 13.89%) (Table 2).

**Fig. 1:**
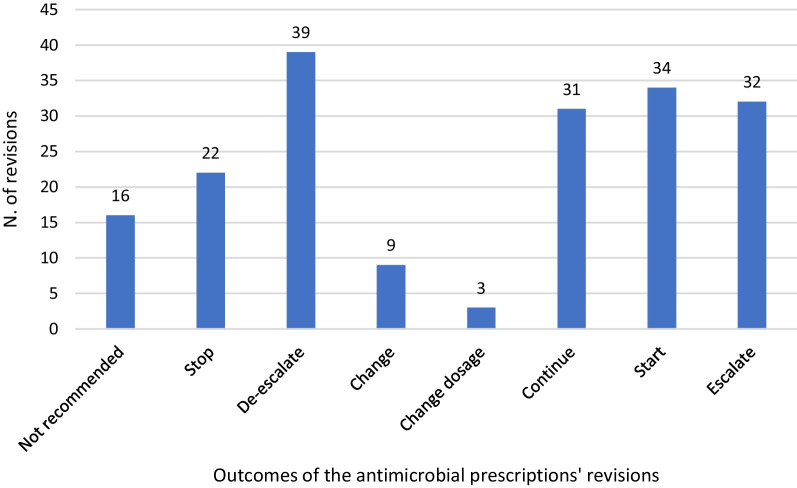
Outcomes of the antimicrobial prescriptions’ revisions

Regarding the outcomes of the prescriptions’ revisions, those bringing to a de-escalation of an ongoing antimicrobial therapy were the most prevalent (39 revisions, 20.97%), followed by decisions to start an antimicrobial therapy (34 revisions, 18.28%) and to escalate (32 revisions, 17.20%). Antimicrobial treatments were left unmodified in 31 revisions (16.67%) and were discontinued or not recommended in 22 and 16 revisions, respectively (11.83% and 8.60%). Finally, antimicrobial choices and dosages were modified in 9 and 3 revisions (4.84% and 1.61%, respectively) (Fig. 1). As a result, antimicrobials were discontinued, not prescribed or de-escalated in 77 revisions (41.4%).

### Occurrence of MRDO isolates

Comparing Period A and Period B, we reported an overall decrease of the total occurrence of MDRO isolates, although it did not reach a statistically significance. Among carbapenem-resistant pathogens, only *Pseudomonas aeruginosa* isolates showed a statistically significant decrease (p-value 0.003, Table 3)*.* Occurrence of ESBL-producing enteric Gram-negative bacteria also decreased but not significantly. Remarkably, during the intervention period no *Clostridioides difficile* infections were reported.

**Table 3:** Summary of the study outcomes

	Period A (1st July 2017–30th June 2018)	Period B (1st July 2018–30th June 2019)	p-value
Patients admitted for at least 48 h, n	709	698	0.67
Occurrence of MDRO infections			
Total MDRO isolates, n	53	39	0.22
Carbapenem-resistant *Acinetobacter baumannii*, n	0	1	0.25
Carbapenem-resistant *Klebsiella pneumoniae*, n	7	6	0.42
Carbapenem-resistant *Pseudomonas aeruginosa,* n	7	1	< 0.01
*Clostridioides difficile,* n	2	0	0.10
ESBL-producing enteric Gram-negative bacteria, n	18	14	0.59
Methicillin-resistant *Staphylococcus aureus*, n	19	16	0.37
Vancomycin-resistant enterococci, n	2	1	0.60
Length of in-hospital stay, mean	9.46	9.8	0.60
All-cause in-hospital mortality, n (%)	19 (2.68)	15 (2.15)	0.50
Antibiotic consumption			
Carbapenems, DDDs*100 PDs, mean	4.53	1.51	0.01
Daptomycin, DDDs*100 PDs, mean	2.64	0.05	< 0.01
Clindamycin, DDDs*100 PDs, mean	0.33	3.34	< 0.01
Antibiotic costs, euros	54,876.44	21,777.26	0.03

*ESBL* extended-spectrum beta-lactamase

*DDDs: Defined Daily Dose

### Secondary outcomes

No statistically significant changes before and after the implementation of the ASP in the Vascular Surgery ward were observed in terms of amount of admittances, length of stay and all-cause in-hospital mortality (Table 3).

During the 12-month period following the start of the ASP, we noticed an improvement in the antimicrobial prescription appropriateness. More precisely, our intervention was particularly effective in reducing the administration of carbapenems, daptomycin and linezolid. The monthly mean DDDs*100 PDs of carbapenems decreased from 4.53 to 1.51 (p-value 0.01) and daptomycin DDDs*100 PDs from 2.64 to 0.05 (p-value < 0.01, see Table 3). Linezolid DDDs*100 PDs also diminished from 0.49 to 0.26 (p-value 0.43, data not shown).

Glycopeptides, specifically vancomycin, were used as anti-MRSA agents in place of daptomycin, so we noticed a non-statistically significant increase of administered DDDs*100 PDs in concomitance with our intervention (p-value 0.14, data not shown). Similarly, consumptions of both trimethoprim/sulfametoxazole (TMP/SMX) and fluoroquinolones increased non significantly: TMP/SMX DDDs increased from 3,953 to 7,191 (p-value 0.0584, data not shown) whereas fluoroquinolones DDDs increased from 9,1893 to 13,408 (p-value 0.2553, data not shown). Oral fosfomycin was introduced in the clinical practice of the Vascular Surgery ward to treat uncomplicated lower urinary tract infections, in place of beta-lactams. In particular, DDDs*100 PDs of fosfomycin increased from 0 to 0.65 (p-value 0.0258, data not shown).

Penicillins had a non-significant increase of administration, from 33,92 to 36.91 DDDs*100 PDs (p-value 0.9372, data not shown), as third generation cephalosporins, which moved from 3,52 to 4,29 DDDs*100 PDs (p-value 0.6649, data not shown). As regards antifungals, we noticed a non-significant decrease of DDDs*100 PDs from 0.23 to 0.15 (p-value 0.96, data not shown).

During the intervention period, a significant reduction in antimicrobial costs was observed. In particular, the total cost of antimicrobial drugs prescribed in the Vascular Surgery ward after the start of the ASP was equal to 21.777,26 €, with a net difference of 33.099,18 € (60,31%; p value 0.03, see Table 3) compared to the previous 12 months (54.876,44 €).

## Discussion

After performing a 12-month period of antimicrobial stewardship intervention in the Vascular Surgery ward of our hospital, we have certainly achieved some positive results, both from a microbiological and economic perspective. On the one hand, in Period B we reported a minor occurrence of carbapenem-resistant *Pseudomonas aeruginosa* isolates and the absence of infections caused by *Clostridioides difficile*. On the other hand, we also observed a significant reduction in the consumption of some broad-spectrum and expensive antimicrobial drugs compared to Period A, with no adverse influence on LOS and mortality. These findings are in line with the avowed concept of the utility of ASPs in minimizing antimicrobial resistance and *Clostridioides difficile* diarrhoea [21]. Hence, since the strong correlation between the multidrug-resistant *Pseudomonas aeruginosa* occurrence and broad-spectrum antimicrobial consumption, a substantial decline in the prescription of these drugs, which is accomplished by ASPs, might effectively control the occurrence of such resistant microorganism [22].

Regarding antimicrobial costs, a recent narrative review thoroughly discussed the most recent evidence on the positive consequences of ASPs for healthcare systems, concluding that, despite ASPs would be cost-effectiveness, the majority of the available studies do not deal with cost–utility analyses, rendering an unambiguous evaluation difficult [23]. In our particular case, though, we have to consider that the model of stewardship intervention we implemented has not required any further costs. In fact, our ASP can be classified as a “handshake stewardship” strategy, as it is centered on the in-person approach to feedback and thus it is based on the commitment of the two ID consultants in terms of number of hours spent weekly in the surgical ward [24].

Along with considering the achieved results as a prompt to reflect on the magnitude and apparent simplicity of ASPs the strength of our work resides in laying the groundwork for a team effort. In fact, we believe that a successful ASP cannot be indeed achieved without the support and joint collaborative effort between different hospital units; this is favoured by the direct communication promoted by a model of stewardship as ours, which includes some of the core elements required for an ASP, namely multidisciplinary teamwork, accountability, enabling actions and education [25].

It could be argued that, despite our work has been conceived as a quality-improvement study, no changes have been observed in terms of LOS and all-cause in-hospital mortality, which are part of the so-called outcome measures. However, it has been recognised that these kinds of indicators may be misleading in evaluating the quality of hospital care; instead, clinical process measures, such as the formerly mentioned decline in occurrence of healthcare-associated infections, must be preferred, as they are a direct measure of performance based on adherence to established clinical standards [26]. In fact, in our study a solid effect on these measures has been reported, so we can assert with good reason that an improvement in the quality of care of the Vascular Surgery ward has been obtained thanks to the implementation of the ASP.

We should not neglect some of the limitations of our study. Firstly, the retrospective nature of the data collected for Period A may have influenced the outcome, since unrecognised confounders cannot be excluded. Secondly, at the time the study was conducted the Pharmacy Unit of our Hospital lacked a computerized drugs prescription and administration system. For this reason, the data of antimicrobial consumption were inferred from the amount dispensed from the Pharmacy Unit to the Vascular Surgery ward and thus may not accurately reflect the real usage trend. Thirdly, we did not analyse the extent of antimicrobial prescriptions following the patients’ discharge. These data are a significant marker for antimicrobial exposure and may impact both colonization and infections caused by MDRO.

## Conclusions

In conclusion, the implementation of a 12-month antimicrobial stewardship program brought significant clinical and economic results in the Vascular Surgery ward of our hospital, highlighting the benefits of a multidisciplinary teamwork and the importance of the commitment of each actor involved, both medical and non-medical.

We hope to further extend this model of antimicrobial stewardship program to a hospital level, possibly integrating it with modern technologies intended to help the prescription activity and the collection of data for additional evaluations.

## Data Availability

The datasets used and/or analysed during the current study are available from the corresponding author on reasonable request.

## Abbreviations

ABSSSI: Acute bacterial skin and skin structure infection
ADs: Antimicrobial drugs
AMR: Antimicrobial resistance
ASP: Antimicrobial stewardship programs
CI: Confidence interval
DDD: Defined daily dose
DFI: Diabetic foot infection
EANM: European Association of Nuclear Medicine
ESBL: Extended spectrum beta-lactamase
ESCMID: European Society of Clinical Microbiology and Infectious Diseases
ESNR: European Society of Neuroradiology
FDA: Food and Drugs Administration
CDC: Centers for disease control and prevention
HAP: Hospital-acquired pneumonia
HCV: Hepatitis C virus
HIV: Human immunodeficiency virus
IAI: Intra-abdominal infection
ID: Infectious diseases
IDSA: Infectious Diseases Society of America
IQR: Interquartile range
LOS: Length of stay
MRSA: Methicillin-resistant *Staphylococcus aureus*
MDRO: Multidrug-resistant organism
PAD: Peripheral artery disease
PPE: Personal protection equipment
SD: Standard deviation
SSI: Surgical site infection
TMP/SMX: Trimethoprim/sulfametoxazole
UTI: Urinary tract infection
WHO: World Health Organization
WSES: World Society of Emergency Surgery
